# My Labor-Saving Husband

**Published:** 1864-12

**Authors:** 


### MY LABOR-SAVING HUSBAND.

HINTS FOR OTHER HUSBANDS.

Some husbands are more plague than profit, and make vastly more work than they do; but mine is one to brag about. When I was married, to my shame be it spoken, I had never made a loaf of bread or a pie. I had no idea of saving time or saving work. But I had a husband who had love enough for me to bear with my simplicity, and not scold when the bread was burned and the pies not fit to eat. Going into the kitchen one morning, he saw me baking buckwheat cakes, and greasing the griddle with a piece of pork on the end of a fork. He said nothing, but went into the wood-house, and soon returned with a smoothly-whittled stick, about six inches long, through the split end of which he had passed a folded strip of white cloth, and then wound it around the end and tied it with a bit of string. So I had a contrivance which could be dipped in melted grease, and put it smoothly over the griddle.

One day he saw me scouring knives with a piece of cloth. "Dear me!" said he, "you will surely cut your fingers." So he contrived a machine by nailing a broad piece of cork to a spool for a handle, sinking the head of the nail into the cork so far that it should not touch the knife. This lifts the hand from the knife and does not cramp the fingers.

I used to call him occasionally to thwack over the heavy mattress and straw bed for me. "What a nuisance!" he exclaimed, and so replaced them by a spring mattress. Of all the nice things for beds, this is the best. It is always in place, requires no shaking up, and it takes only three minutes to replace the bed-clothes, and the bed is made. It always looks round and inviting, and gently yields to the weight of the sleeper.

He saw the dish-towels hanging helter-skelter around the kitchen stove, and forthwith made the most convenient hanging-frame, over the wood-box, where it can take up no room and is near the stove. Here the towels hang smoothly and are always in place.

I fretted because my refrigerator had no shelves, and I could not make room enough for all the meat, butter, and milk. So he made two racks, and fitted ventilated shelves from the one to the other. The shelves are ventilated by being bored thick with auger-holes, and can be removed for scrubbing.

He is troubled to see me sew, sew, and stitch, stitch, and makes sewing-machines the constant topic of conversation. He reads to me every advertisement and every letter from women who praise them in the papers. If he could make one, I should be in possession of one immediately; but as he cannot, I must wait till "the ship comes in." These are some of the ways by which he lightens the labor of the house. Would more husbands were like him. Perhaps, another time, I shall tell you how he contrives his own garden tools, and saves time and money by his ingenuity.

